# Markers of Carotid Plaque Destabilization in Patients With Sleep-Disordered Breathing

**DOI:** 10.3389/fneur.2022.811916

**Published:** 2022-02-16

**Authors:** Lena Lavie, Erez Si-On, Aaron Hoffman

**Affiliations:** ^1^Unit of Anatomy and Cell Biology, The Ruth and Bruce Rappaport Faculty of Medicine, Technion-Israel Institute of Technology, Haifa, Israel; ^2^Department of Vascular Surgery and Transplantation, Rambam Health Care Campus, The Ruth and Bruce Rappaport Faculty of Medicine, Technion-Israel Institute of Technology, Haifa, Israel

**Keywords:** sleep disordered breathing, carotid plaque, nitro-oxidative stress, inflammation, 3-nitrotyrosine (3-NT), smooth muscle cell-actin, lipids, plaque vulnerability

## Abstract

Sleep-disordered breathing (SDB) is a nightly respiratory condition characterized by intermittent hypoxia, leading to oxidative stress, inflammation, and atherosclerosis. However, most cellular markers of human carotid plaques in SDB have not yet been assessed. We aimed at characterizing the cellular, inflammatory, and nitro-oxidative stress markers in carotid plaques obtained from 25 patients undergoing endarterectomy and screened for SDB. Sleep studies were performed during their preoperative hospitalization night using the Watch-PAT 100 device. Oxygen desaturation index (ODI) was used for dividing patients into two groups. Fourteen patients with ODI >5 were designated as SDB and 11 patients with ODI ≤ 5 as non-SDB. Demographics, comorbidities, cardiovascular risk factors, and medications were recorded. Cellular markers in plaques were analyzed by immunofluorescence using confocal microscopy. The expression of neutrophils was identified by CD66b+ and neutrophil elastase, macrophage-foam cells were identified by CD163+, and scavenger receptors by CD68+ and CD36+ expression. Additional markers included 3-nitrotyrosine, endothelial CD31, and smooth muscle cell-actin (SMC-actin). Plaques' lipids were determined by immunohistochemistry with Oil Red O staining. Notably, significantly higher values were found for SDB as compared to patients with non-SDB for 3-nitrotyrosine (*p* <0.004) and intracellular lipids' content (*p* <0.02), whereas SMC-actin was lower (*p* <0.006). There were no significant differences between patients with carotid-associated symptoms (symptomatic) and patients without carotid-associated symptoms (asymptomatic). However, a sub-group of symptomatic patients with co-existent SDB expressed the highest 3-nitrotyrosin, and intracellular lipids levels, and the lowest SMC-actin levels, whereas non-SDB/asymptomatic patients expressed the lowest 3-nitrotyrosin and lipids levels and the highest SMS-actin levels among all patients. Accordingly, ODI was lowest in non-SDB/asymptomatic patients and highest in SDB/symptomatic. In conclusion, plaques of patients with SDB were characterized by markedly increased levels of 3-nitrotyrosine and intracellular lipids content. Conversely, SMC-actin levels were significantly lower. These three markers, such as increased 3-nitrotyrosine and intracellular lipids and decreased SMC-actin are associated with plaque vulnerability and instability. These findings are in line with earlier reports demonstrating increased intima-media thickness in large cohorts of sleep apnea and patients with SDB, and thus, may indicate a higher susceptibility to plaque vulnerability and rapture in patients with SDB.

## Introduction

Obstructive sleep apnea (OSA) or sleep disordered breathing (SDB)—its non-symptomatic manifestation—is a nightly respiratory condition characterized by recurrent oropharyngeal upper airway collapse during sleep leading to multiple cycles of hypoxic episodes followed by blood reoxygenation. While blocking the respiratory system in OSA provokes hypoxic events, resuming respiration induces the reoxygenation phase. This intermittent respiration is exhibited as multiple cycles of hypoxia and reoxygenation throughout the night, resulting in intermittent hypoxia (IH). Moreover, this intermittent respiration directly affects blood reoxygenation/deoxygenation levels, and greatly affects physiological, biochemical, and molecular pathways, such as oxidative stress, inflammation, and sympathetic activation, leading to the development of atherosclerosis (1, 2).

The prevalence of symptomatic OSA with characteristic complaints, such as hypersomnolence, is high in the general population. In moderate to severe OSA with an apnea-hypopnea index (AHI) ≥15, the prevalence is 10% in men and 3% in women aged 30–49 years, rising to 17% among men and 9% among women of 50–70 years old (3). However, the prevalence of asymptomatic non-somnolent patients with SDB is higher, affecting as much as 24% of men and 9% of women (4). In selected populations, such as obese or elderly, it may rise to 60% (5), and in patients with acute myocardial infarction (MI), SDB is higher than 60% (6). In patients with stroke, at least 50–70% might be affected (7, 8). This association is stronger in men and is linked with a higher mortality rate and worse outcomes following stroke (9, 10).

A large number of clinical prospective and epidemiological studies demonstrated that cardiovascular and cerebrovascular associated risk factors, such as hypertension, hyperlipidemia, hyperglycemia, and sympathetic activation are increased in OSA (11). Thus, the impact of OSA on the cardiovascular and cerebrovascular system has been well-documented over the past decades and it has become a recognized cardiovascular and cerebrovascular risk factor (12). OSA is now implicated in the etiopathogenesis of stroke, coronary artery disease, and congestive heart failure. The putative mechanisms involved in the pathogenesis of cardio/cerebrovascular disease in OSA include oxidative stress, fibrinolytic imbalance, inflammation, endothelial dysfunction, and atherosclerosis (1, 12).

Atherosclerosis is a progressive disease characterized by the accumulation of inflammatory cells, lipid deposits, and fibrous elements in the large arteries, and is considered a chronic inflammatory response. This inflammatory response is associated with the activation of an array of cellular components, such as the endothelium, leukocytes, platelets, and intimal smooth muscles cells. Epidemiological studies over the past 40 years identified a great number of risk factors for atherosclerosis. Among them are age, gender, obesity, cigarette smoking, hypertension, diabetes mellitus, plasma homocysteine, and serum cholesterol. Hence, OSA has been implicated as one of the risk factors inducing atherosclerosis (13, 14). Cross-sectional studies have shown consistently that OSA is independently associated with surrogate markers of premature atherosclerosis. For instance: Suzuki et al. (15) reported that the severity of OSA was independently related to atherogenic thickening of the carotid artery wall as measured by ultrasonic evaluation of intima-media thickness (IMT). Baguet et al. (16) showed that the severity of oxygen desaturation predicted carotid IMT and plaque occurrence in patients with OSA without a known cardiovascular disease (CVD). Using ultrasound, the coronary atherosclerotic volume was shown to be greater in patients with AHI above 15 events/h, compared to patients with an AHI below 15. Moreover, the coronary atherosclerotic plaque (17) and its IMT were significantly positively correlated with AHI severity (18). In a recent large cohort of 2009 participants, OSA was independently associated with the increased carotid IMT in a dose-response relationship, and this association was partially mediated by inflammation and dyslipidemia (19). Also, treatment with continuous positive air pressure (CPAP) which is an effective treatment of OSA, significantly improved early signs of atherosclerosis, such as IMT and pulse-wave velocity (20). Furthermore, in a randomized clinical trial, the treatment of OSA with CPAP was shown to attenuate carotid atherosclerosis (21). Also, the IH associated with OSA increased sympathetic nerve activity. This may exacerbate the systemic inflammation caused by oxidative stress, and may further contribute to an increase in the size of carotid IMT in OSA (22). In a study by Gunnarsson et al., investigating 689 participants from The Wisconsin Sleep Cohort of community dwelling subjects, minimum oxygen saturation (SaO_2_) was found to be an independent risk factor for increased future plaque burden (23). In a study by Zaho et al. investigating 1,615 participants from the Multi-Ethnic Study of Atherosclerosis has shown that OSA, defined as AHI ≥15, was significantly associated with the presence of subclinical carotid atherosclerosis in men and women younger than 68 years (24). These latter findings are clinically highly relevant since carotid plaque burden is a strong predictor of cardiovascular (CVD) events.

Due to the high prevalence and the worse outcomes of stroke in patients with OSA/SDB (3, 10, 24), we hypothesized that atherosclerotic markers in carotid plaques of patients with SDB might be increased or altered. Thus, we investigated some biochemical and cellular markers in carotid plaques obtained from patients undergoing endarterectomy and compared them between patients with and without SDB. We determined the presence of several inflammatory cells, such as macrophages, neutrophils, and foam cells, the nitro-oxidative stress marker 3-nitrotyrosine (3-NT), lipid content in the plaques, the presence of vessels (CD31), and smooth muscle cells (SMCs) utilizing smooth muscle cell-actin (SMC-actin) as a marker. These markers have not been studied, thus, far in this context.

## Materials and Methods

### Patients

A total of 25 carotid plaque samples were obtained from patients undergoing carotid endarterectomy with carotid stenosis of 50% or more in symptomatic patients and 70% or more in asymptomatic patients. Patients were hospitalized in the Department of Vascular Surgery and Transplantation, Rambam Medical Center, Israel. Demographic parameters were recorded, and Sleep Studies were performed during their preoperative hospitalization night using the Watch-PAT 100 device. This device was extensively validated against polysomnographic recordings for the diagnosis of OSA/SDB (25, 26). Data were analyzed to determine oxygen desaturation index (ODI) (number of desaturations >4% events/h) that were calculated in terms of events per hour, AHI, respiratory disturbance index (RDI), mean oxygen (Mean Oxy.), and minimal oxygen (Min. Oxy.). Based on the sleep study, patients were stratified according to ODI into 2 groups. Fourteen patients with ODI >5 were designated as carotid plaque—SDB and 11 patients with ODI ≤ 5 were designated as a non-SDB group. The protocol was approved by the Local Humans Rights Committee of RAMBAM Medical Center (RMB-0175-14), according to the declaration of Helsinki, and all patients signed an informed consent form.

### Preparation of the Specimens

Carotid plaque arteries were removed using standard surgical techniques and with minimal manipulation to the specimen. Carotid plaque tissues were obtained immediately after surgery and were stored in phosphate buffered saline (PBS) solution (4°C). The specimens obtained were embedded into an optimum cutting temperature (OCT) compound (LEICA, 020108926) and stored at −80°C for further analysis by immunohistology and immunohistochemistry by using the confocal microscopy.

Mouse monoclonal primary antibodies were used to identify neutrophils (anti-CD66b) and macrophage-foam cells (anti-CD163). Rabbit primary polyclonal antibodies used for double labeling the cells with additional markers included the scavenger receptors CD68, CD36, CD31, 3-nitrotyrosine, and SMC-actin. Intra/extracellular lipids were determined by immunohistochemistry with Oil Red O staining.

### Immunohistochemistry and Immunofluorescence

For histochemical and immunohistochemical analyses, frozen sections were cut at a thickness of 5 μm and mounted on microscope slides. For histochemical analysis, the 5-μm-thick sections were stained with H&E and lipid deposits in the plaques were detected using Oil Red O staining (27, 28).

Primary antibodies were diluted to 1:100, and a Histostain-Plus Kit AEC, Broad Spectrum (Invitrogen, MA, USA) was used for their detection. Sections were incubated with primary antibodies for 2 h at 37°C in an incubator. Then, sections were incubated with secondary antibodies from the Histostain-Plus kit, for 30 min at 37°C. The 3-amino-9-ethylcarbazole (AEC) was used as a chromogen to detect the antibodies. The following cell populations were identified and quantified in the carotid body plaques: polymorphonuclear cells (PMNs) by CD66b and neutrophil elastase (NE), the scavenger receptors CD36, CD68 for foam cells, and macrophages were identified by CD163 and CD68. Additional markers included antibodies against the oxidative stress marker-3-nitrotyrosin and CD31—for vessel identification by the presence of endothelial cells and smooth muscle actin (SM-actin), a marker of arterial wall remodeling.

For immunofluorescence analysis, slides were fixed with acetone for 15 min at 4°C and washed with PBS. After blocking with 10% normal goat serum in RPMI-1640 medium, slides were incubated overnight at 4°C using primary antibodies. Mouse monoclonal primary antibodies included: anti-CD66b (80H3, AbD Serotec, Oxford, UK), anti-CD163 (GHI/6), anti-3 nitrotyrosine (3-NT), and anti- SMC-actin (Santa Cruz Biotechnologies, Inc., CA, USA). Rabbit primary polyclonal antibodies included: anti-CD68 (ProteinTech, IL, USA), anti-CD36 (SR-B3, Novus, MO, USA), anti-neutrophil elastase (NE) (Calbiochem, San Diego, CA, USA), and anti-CD31 (ab32457, abcam, UK). Then, the cells were washed and incubated with 1/400 secondary antibodies Cy2 (CF 488A)-conjugated goat anti-rabbit IgG and/or Cy5 (CF 647)-conjugated goat anti-mouse IgG (Biotium, Hayward, CA, USA). Isotype controls included purified mouse IgG1 (clone MG1-45) and IgG2 (clone MOPC-173, BioLegend, San Diego, CA, USA), and rabbit IgG (Santa Cruz Biotechnologies, Santa Cruz, CA, USA). After washing, slides were mounted with a mounting medium containing 4', 6-diamidino-2-phenylindole (DAPI) for nuclear staining (Vectashield H-1000, Vector lab. Inc. Burlingame, CA, USA). Slides were analyzed by a confocal laser scanning system (LSM 700) using Nikon E600 (Japan) fluorescence microscope and Plan Apo ×40 immersion oil objective. Fluorescent intensities were integrated with Image J software (Wayne Rasband, NIH, MD, USA). Mander's Overlap Coefficient (MOC) was used to quantify co-localization (29). Histological observations were recorded by an observer who was blinded to the clinical information.

### Statistical Analysis

Data are expressed as a mean ± SD or as a mean ± SE. Continuous variables were presented as means and SEs and categorical variables are presented as absolute numbers and percentages. Two-tailed *t*-tests for independent samples were used for between-group comparisons of parametric data, while chi-square or Fisher's exact test were used for the comparison of frequencies. Pearson's correlation analysis was used to explore the association between variables. The value of *p* <0.05 was considered significant.

## Results

### Patients' Demographics, Sleep Data, Comorbidities Risk Factors, and Medications

Table 1 presents the demographics, sleep data, comorbidities and risk factors, and the use of medications for the 25 patients investigated. The sleep data are presented as the average values ± SD for ODI, RDI, and AHI, as well as for Min. Oxy. and Mean oxy. Additionally, their medians and the range for each parameter in each study group are presented. According to the sleep studies, based on IH severity, 11 patients were designated as non-SDB (ODI ≤ 5, 1.5 ± 0.5; 95% *CI*; 1.5 ± 0.3) and 14 patients as SDB (ODI >5, 16.1 ± 15.4; 95% *CI*; 16.1 ± 8.1) (*p* <0.003). Out of the 14 patients with SDB, 12 were mild to moderate, with ODI values ranging from 6.1 to 18.8 events/h, while only two patients had severe SDB with ODI of 33.8 and 63.1 events/h. As can be seen in Table 1, there were also significant between-group differences for AHI and RDI. The patients with non-SDB included 6 women and 5 men while the SDB group consisted of 12 men and 2 women. Although not statistically significant, the prevalence of women with plaques in the SDB group was lower than in men as previously described (30, 31).

**Table 1:** Demographics, sleep data, comorbidities, cardio-cerebrovascular risk factors, and medications in patients with sleep-disordered breathing (SDB) and non-SDB.

**Variables**	**Non-SDB**	**SDB**	**Significance - *p*<**
Total *N* = 25	11	14	
Age (years)	69.8 ± 8.6	72.4 ± 9.8	ns
BMI (Kg/m^2^)	25.8 ± 4.1	28.9 ± 2.7	0.04
Gender	5M/6F	12M/2F	ns^*^
**SDB measures**			
ODI (event/hour)	1.5 ± 0.5	16.1 ± 15.4	0.003
Median, (Range)	1.9, (0.0–4.8)	11.5, (6.1–63.0)	
RDI (event/hour)	8.6 ± 5.3	27.5 ± 21.4	0.0085
Median, (Range)	7.6, (1.3–20.0)	19.8, (12.1–90.0)	
AHI (event/hour)	5.0 ± 3.5	24.8 ± 21.7	0.0067
Median, (Range)	5.4, (0.4–10.9)	18.4, (11.2–89.7)	
Min. Oxy.	87.0 ± 7.2	82.2 ± 5.3	ns
Median, (Range)	89.0, (72.0–92.0)	82.0, (70.0–88.0)	
Mean Oxy.	93.9 ± 3.0	92.5 ± 2.6	ns
Median, (Range)	94.0, (86.0–98.0)	93.0, (86.0–95.0)	
**Comorbidities and risk factors -** ***N*** **(%)**			
Symptomatic	5 (45.4)	6 (42.8)	ns
Asymptomatic	6 (54.5)	8 (57.1)	ns
IHD	4 (36.4)	5 (35.7)	ns
sp/MI	3 (27.3)	4 (28.6)	ns
CVA	7 (63.6)	8 (57.1)	ns
TIA	2 (18.2)	4 (28.6)	ns
HTN	9 (81.8)	14 (100.0)	ns
PVD	4 (36.4)	7 (50.0)	ns
DM	7 (63.6)	8 (57.1)	ns
COPD	4 (36.4)	6 (42.8)	ns
Hyperlipidemia	8 (72.7)	13 (92.8)	ns
**Smoking status -** ***N*** **(%)**			
Current	5 (45.4)	6 (42.8)	ns
Non-smoking	4 (36.4)	6 (42.8)	ns
Past smoking	2 (18.2)	2 (14.3)	ns
**Drugs—*****N*** **(%)**			
Aspirin	7 (63.6)	10 (71.4)	ns
Plavix	4 (36.4)	10 (71.4)	ns
Clex	3 (27.3)	0 (0.0)	ns
Statins	8 (72.7)	13 (92.8)	ns
Ace inhibitors	7 (63.6)	8 (57.2)	ns
Ca channel blockers	5 (45.4)	7 (50.0)	ns
β blockers	5 (45.4)	4 (28.6)	ns
α blockers	1 (9.1)	4 (28.6)	ns

*Values are presented as average ± SD, where appropriate. IHD, ischemic heart disease; s/p MI, status post myocardial infarction; CVA, stroke or cerebrovascular accident; TIA, transient ischemic attack; HTN, hypertension; PVD, peripheral vascular disease; DM, diabetes mellitus; COPD, chronic obstructive pulmonary disease*.

* *Gender differences were not statistically different using Fisher's exact test*.

As expected, there were significant differences in sleep measures affected by SDB between the two patient groups except for Mean Oxy. and Min. Oxy. which tended toward significance (*p* <0.085). These non-significant differences might be attributed to the high prevalence of chronic obstructive pulmonary disease (COPD) in both groups (36.4 and 42.8% in non-SDB and SDB, respectively, and 40% of total patients). High COPD prevalence in patients with carotid plaques was also described in earlier studies (31). The only significant difference between patients with SDB and non-SDB was in body mass index (BMI) (26.0 ± 4.0 vs. 29.3 ± 4.0, *p* <0.04). All other measures, such as comorbidities risk factors, smoking status, and drugs, did not differ significantly between the study groups.

### Cellular Inflammatory and Oxidative Stress Markers in Plaques of Patients With SDB and Non-SDB

The plaques were quantitatively analyzed for cellular, inflammatory, and oxidative stress markers as depicted in Table 2. The data are presented by the mean (%) of the stained area of at least 3 different sections from the center of each plaque for each marker. In each section, at least three different fields were analyzed for each of the various markers determined. There was a statistically significant between groups difference in **3-NT** (0.004) which was 4.7-fold higher in SDB as compared with non-SDB patients. Similarly, the **intracellular lipid** content in the plaques of patients with SDB was significantly 2-fold higher (*p* <0.02), although the expression of foam cells did not statistically differ between the groups. In contrast, the expression of SMC-actin, representing SMCs and vascular remodeling, was significantly 4.9-fold lower (*p* <0.006) in the SDB patients' group. Higher 3-NT levels and a higher content of lipids on one hand and a lower SMC-actin on the other might indicate that patients with SDB are at a higher risk for plaque atherogenicity and instability than patients with non-SDB. In addition, neutrophil elastase tended to be significantly higher (*p* <0.07) in patients with SDB. Except for these main differences between patients with SDB and non-SDB, there were no other significant differences between the groups in the cellular parameters investigated. Interestingly, although not statistically significant, CD31—a marker that represents neovasculogenesis and is prevalent in unstable plaques (32) was 2-fold higher in patients with SDB.

**Table 2:** Quantitative analysis of cellular plaque markers (by % stained area ± SD) in patients without and with SDB.

**Cellular markers (Stained area, %)**	**Non-SDB plaques**	**SDB plaques**	**Significance-p <**
Neutrophils-CD66b+	0.56 ± 0.93	0.77 ± 1.06	ns
Neutrophil Elastase (NE)	0.51 ± 0.62	1.33 ± 1.30	ns (0.07)
Macrophages-CD163+ (poly)	1.45 ± 2.08	1.54 ± 1.23	ns
Macrophages-CD163+ (mono)	0.42 ± 0.45	1.02 ±1.40	ns
**Scavenger receptors**			
CD68+	1.39 ± 1.45	1.43 ± 1.76	ns
CD36+	2.15 ± 2.11	2.70 ± 2.18	ns
**Neutrophil-derived foam cells**			
CD66b+/CD68+	0.51 ±1.14	0.17 ± 0.38	ns
CD66b+/CD36+	0.16 ± 0.38	0.05 ± 0.11	ns
CD66b+/CD163+	0.01 ± 0.01	0.31 ± 0.55	ns
**Macrophage-derived foam cells**			
CD163+/CD68+	0.06 ± 0.11	0.32 ± 0.62	ns
CD163+(mono)/CD163+(poly)	0.12 ±0.26	0.14 ± 0.19	ns
3-Nitrotyrosine (3-NT)+	**1.29** **±1.07**	**6.11** **±5.10**	**0.004**
3-NT/NE	0.14 ± 0.39	0.82 ±1.48	ns
**Lipids (all)**	13.73 ± 5.25	16.34 ± 6.45	ns
Lipids (intra-cellular)	**2.15** **±1.69**	**4.04** **±2.16**	**0.02**
Lipids (extra-cellular)	8.18 ± 4.04	10.08 ± 6.3	ns
Lipids (crystals)	4.47 ± 2.94	3.85 ± 3.11	ns
Vessels (CD31)	0.81 ± 0.47	1.90 ± 2.78	ns
SMC-actin	**3.67** **±1.65**	**0.74** **±0.55**	**0.006**

*Data are reported as the mean ± SD. The values of p were determined using Student's t-test. (Poly), polyclonal antibody; (mono), monoclonal antibody. Signifucant values and their p values are in bold*.

In Table 3 we performed a Pearson's correlation analysis between ODI and 3-NT across all patients and then separately for men and women, which confirmed the above analysis for 3-NT expression in plaques (all patients *p* <0.001, only men *p* <0.05, and only women *p* <0.00006).

**Table 3:** Correlations between plaque % stained area of 3-nitrotyrosine (3NT) vs. oxygen desaturation index (ODI).

**Correlations (% of stained area)**	***R***	***N***	**Significance-*p*<**
3-NT vs. ODI - all patients	0.61	25	0.001
3-NT vs. ODI - all men	0.48	17	0.05
3-NT vs. ODI - all women	0.97	8	0.00006

*R, correlations; N, number of patients*.

### Cellular Inflammatory and Oxidative Stress Markers in Asymptomatic vs. Symptomatic Patients' Plaques

Patients undergoing endarterectomy are identified as symptomatic or asymptomatic for the presence of carotid plaques. Symptomatic patients are those who had either an ipsilateral cerebrovascular accident (CVA) or a transient ischemic attack (TIA) or amaurosis fugax, in the last 180 days. Asymptomatic carotid artery stenosis refers to stenosis in persons without a history of ischemic stroke, TIA, or other neurologic symptoms referable to the carotid arteries. Comparing the cellular, inflammatory, and nitro-oxidative stress markers, regardless of ODI between patients that were clinically symptomatic to the presence of carotid plaques, and asymptomatic patients, having no clinical symptoms regarding their carotid status (defined by NASCET-North American Symptomatic Carotid Endarterectomy Trial) (33), revealed no statistically significant differences between the groups (Table 4). Except for the expression of neutrophil elastase that was nearly significantly higher (*p* <0.059), while scavenger receptor CD36 expression was nearly significantly lower (*p* <0.055) in symptomatic patients.

**Table 4:** Quantitative analysis of cellular plaque markers (by % stained area ± SD) in asymptomatic and symptomatic patients.

**Cellular markers (Stained area, %)**	**Asymptomatic-plaques (*N* = 14)**	**Symptomatic plaques (*N* = 11)**	**Significance *p*<**
Neutrophils-CD66b+	0.46 ± 0.82	0.95 ± 1.15	ns
Neutrophil Elastase (NE)+	0.53 ± 0.79	1.46 ± 1.26	ns (0.059)
Macrophages-CD163+ (poly)	1.27 ± 1.75	1.81 ± 1.45	ns
Macrophages-CD163+ (mono)	0.61 ± 1.12	0.94 ± 1.13	ns
**Scavenger receptors**			
CD68+	1.28 ± 1.34	1.58 ± 1.94	ns
CD36+	3.20 ± 2.47	1.55 ± 1.14	ns (0.055)
**Neutrophil-derived foam cells**			
CD66b+/CD68+	0.38 ± 1.03	0.24 ± 0.45	ns
CD66b+/CD36+	0.05 ± 0.08	0.16 ± 0.39	ns
CD66b+/CD163+	0.09 ± 0.25	0.30 ± 0.59	ns
**Macrophage-derived foam cells**			
CD163+/CD68+	0.18 ± 0.54	0.26 ± 0.44	ns
CD163+(mono)/CD163+(poly)	0.16 ± 0.28	0.09± 0.07	ns
3-Nitrotyrosine (3-NT)+	2.98 ± 4.13	5.27 ± 4.89	ns
3-NT/NE+	0.49 ± 1.48	0.56 ± 0.66	ns
**Lipids (all)**	13.99 ± 6.01	16.72 ± 5.86	ns
Lipids (intra-cellular)	2.77 ± 1.99	3.77 ± 2.31	ns
Lipids (extra-cellular)	9.11 ± 5.52	9.42 ± 5.53	ns
Lipids (crystals)	3.66 ± 3.19	4.72 ± 2.75	ns
Vessels (CD31)	1.06 ± 1.11	1.98 ± 3.19	ns
SMC-actin	1.84 ± 1.45	2.39 ± 2.44	ns

*Data are reported as the mean ± SD. The values of p were determined using Student's t-test. (Poly), polyclonal antibody; (mono), monoclonal antibody*.

**Figure 1:**
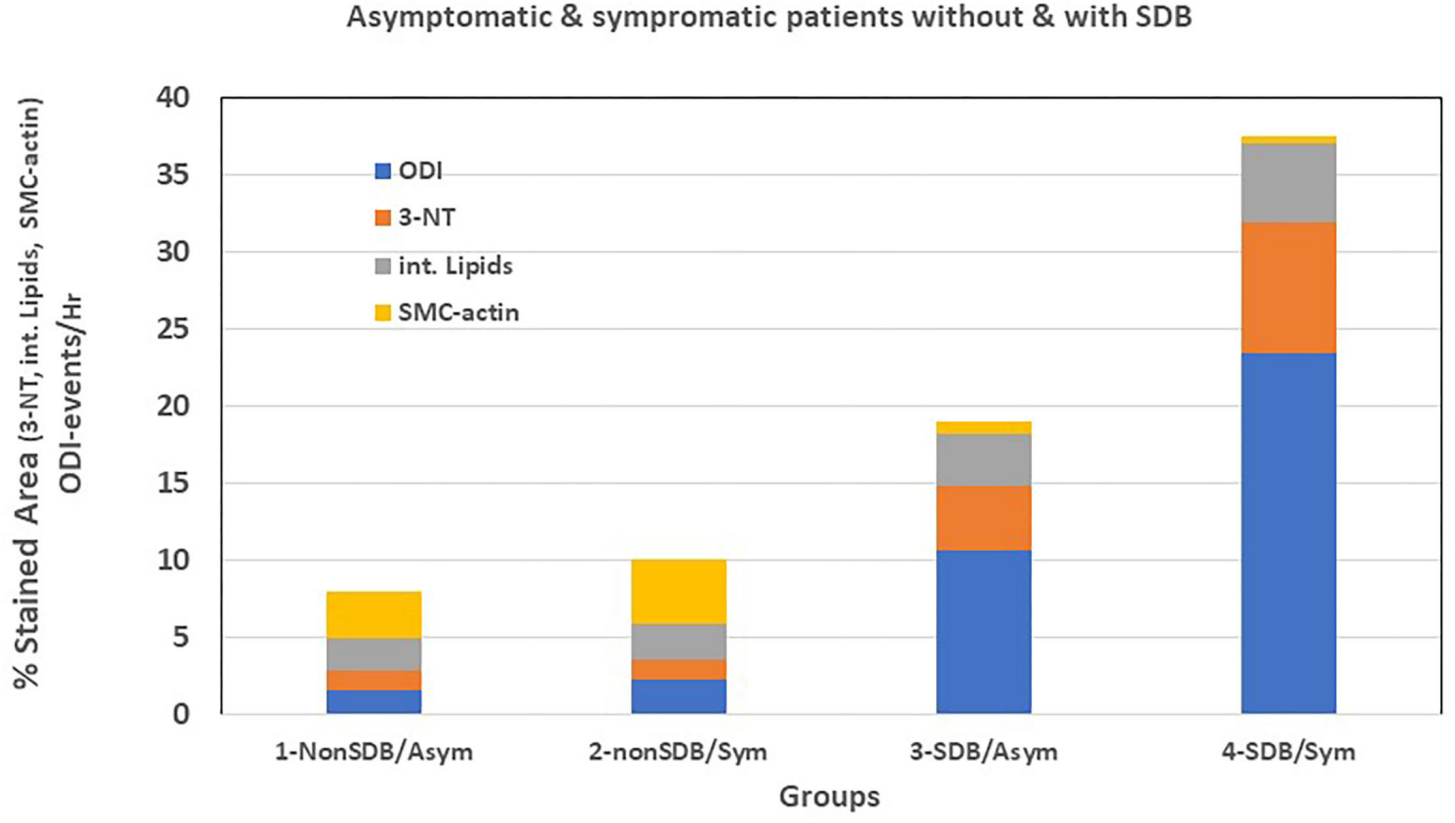
Graphic representation of Table 5.

Moreover, we performed a preliminary analysis to determine if symptomatic patients with SDB show differences in cellular, inflammatory, and nitro-oxidative stress markers, as compared with asymptomatic patients with or without SDB. Thus, patients were divided into four sub-groups: (1) non-SDB/asymptomatic; (2) non-SDB/symptomatic; (3) SDB/asymptomatic; and (4) SDB/symptomatic (Table 5). Although the number of patients in each sub-group is small, patients with SDB, particularly with plaque associated symptoms, showed the highest values for 3-NT and lipids and the lowest values for SMC-actin. In accordance, the average ODI gradually increased from sub-group 1 to sub-group 4. Asymptomatic patients with non-SDB (sub-group 1) had an average ODI of 1.1 ± 0.4 whereas the SDB symptomatic (sub-group 4) had an ODI of 23.4 ± 8.8 events/h. These data suggest that the presence of SDB promotes atherogenic expression in the plaques although an association with being symptomatic cannot be excluded. For convenience and clarity, Figure 1 illustrates these data, emphasizing the contribution of ODI to the increased expression of 3-NT and intracellular lipids and the decrease in SMC-actin.

**Table 5:** All patients were divided into four sub-groups according to presence or absence of SDB and as asymptomatic or symptomatic.

**Group (N)**	**ODI**	**3 Nitro-tyrosine**	**Smooth muscle cell - actin**	**Lipids -intracellular**
1 non-SDB Asymptomatic (6)	1.1 ± 0.4	1.29 ± 0.51	3.09 ± 0.72	2.09 ± 0.65
2 non-SDB Symptomatic (5)	2.2 ± 0.8	1.36 ± 0.38	4.25 ± 0.97	2.24 ±0.80
3 SDB Asymptomatic (8)	10.6 ± 1.9	4.26 ± 1.81	0.90 ± 0.31	3.29 ± 0.78
4 SDB Symptomatic (6)	23.4 ± 8.8	8.59 ± 1.73	0.53 ± 0.25	5.04 ± 0.74

*Cellular plaque components (by % stained area) were statistically analyzed for these markers. N, number of patients in each group. Significance for 3-nitrotyrosine, group 4 vs. group 1, p <0.007; smooth muscle cell–actin (SMC-actin), group 4 vs. group 1, and group 3 vs. group 1, p <0.03; intracellular lipids, group 4 vs. group 1, p <0.01. A smaller number of patients' plaques were analyzed for smooth muscle cell actin—in groups 1, 2, and 4 only plaques of three patients were determined and in group 3, plaques of four patients were determined. Data are presented as average ± SE*.

## Discussion

Atherosclerotic processes are a common denominator in a great number of pathological conditions, such as ischemic stroke, which is a leading cause of disability and economic burden and is responsible for over 10% of mortality worldwide (34). Carotid plaque stenosis, one of the leading causes of ischemic stroke, is a multifactorial and complex process. It involves fundamental mechanisms of atherosclerosis, such as oxidative stress, inflammation, lipid accumulation, angiogenesis, apoptosis, proteolysis, and thrombotic processes which are among the main factors of carotid plaque vulnerability and rupture (34, 35).

Sleep disordered breathing, another risk factor for the occurrence of stroke, is also characterized by atherosclerotic processes (24). Thus, in the current study, we sought to investigate the potential differences in inflammatory and oxidative stress markers between carotid plaques of patients with and without SDB. Several important findings emerged from this study. First, the expression of the nitro-oxidative stress marker 3-NT was significantly higher by nearly 5-fold in patients with SDB. Correlations between ODI and 3-NT were statistically significant across all patients and then separately for men and women, confirming the above analysis. Second, the content of intracellular lipids was significantly higher by 2-fold in the plaques of patients with SDB. Third, SMC-actin was significantly lower by 5-fold in patients with SDB as compared to patients with non-SDB. Fourth, there were no statistically significant differences between symptomatic and asymptomatic patients. Fifth, a sub-group of patients with SDB who were also symptomatic had the highest expression of 3-NT and intracellular lipids and the lowest expression of SMC-actin.

Nitrosative stress and oxidative stress are coupled when a reaction between nitric oxide (NO) and superoxide O_2_^**−**^ occurs, forming the very potent nitrating agent—peroxynitrite anion (ONOO^**−**^). Peroxynitrite can nitrate cellular components and biomolecules, such as proteins, lipids, and DNA. Consequently, endothelial dysregulation and dysfunction may occur due to impaired NO availability, thus promoting plaque instability (36). Nitrating tyrosine in proteins gives rise to 3-NT, a marker for nitro-oxidative stress as well as a marker of plaque instability (36, 37). Conversely, reduced nitrosative stress and restored endothelial nitric oxide synthase (eNOS) function were shown to favor plaque integrity and improve NO homeostasis (36). The vastly increased expression of 3-NT observed in patients with SDB clearly indicates that their carotid plaques are exposed to high nitro-oxidative stress and associated with atherogenesis, and therefore, might be associated with increased plaque instability.

In earlier studies, various markers of oxidative stress were shown to increase in plasma, urine, and exhaled air as well as in oxidized lipids, proteins, and DNA of patients with OSA (2, 38). However, this is the first study to demonstrate increased nitro-oxidative stress in carotid plaques of patients with SDB. Increased 3-NT in SDB also supports the increased IMT and the endothelial dysfunction in SDB as previously described (21). In a study by Cherneva et al., conducted on plaques of non-hypersomnolent patients with OSA and controls, flow mediated dilatation (FMD), and IMT were determined by ultrasonography. While FMD was significantly lower in OSA than in controls (*p* <0.013), IMT was higher, but it did not reach statistical significance. In accord with our findings on plaque 3-NT, they reported that urinary 8-isoprostane, a marker of oxidative stress, was significantly higher in those patients with OSA. However, there was no statistically significant difference between the groups in resistin—a plasma marker of inflammation (39). Similarly, most inflammatory cellular markers we investigated did not differ between the groups, although non-significant trends toward higher levels in the neutrophil elastase (*p* <0.07) and macrophage-derived foam cells (CD163+/CD68+) in patients with SDB were observed.

The 2-fold higher levels of **intracellular lipids'** content in plaques of the patients with SDB also indicate that the plaques of patients with SDB are potentially more atherogenic and vulnerable than plaques of patients with non-SDB. This lipid accumulation is among the main factors of carotid plaque destabilization (35).

The expression of SMC-actin which is a marker of smooth muscle cells and represents remodeling of the arterial wall, and thus, the integrity and stability of the plaque, was significantly lower in patients with SDB. SMCs are considered to have a beneficial role in atherosclerotic plaque stabilization by producing collagen and, thereby contributing to the thickening of the fibrous cap (40). This finding similar to the previous findings on increased levels of 3-NT and intracellular lipids further strengthens the notion that plaques of patients with SDB might be more prone to plaque instability and rupture. Two recent studies conducted on plaques of patients with OSA support this notion. In a prospective study, Olejarz et al. reported that the expression of toll-like receptors (TLRs) and the receptor for advanced glycation end products (RAGE) were significantly increased in carotid plaques of moderate to severe OSA when compared with controls or with mild OSA (41). These receptors play a pivotal role in carotid plaque instability and rupture (42). In another study, Migacz et al. investigated the CD40, CD40L, MCP-1, and MMP-9 atherosclerotic markers of plaque vulnerability. The severity of OSA was significantly associated only with MCP-1. The CD40 was nearly significant (*p* <0.056), suggesting that the CD40/CD40L inflammatory pathway together with MCP-1 may contribute to plaque instability and rupture in OSA (43).

We should note that the expression of the endothelial cell marker CD31, although not statistically significant, was 2-fold higher in plaques of patients with SDB as compared with non-SDB. CD31 represents neovasculogenesis for identifying small microvessels and is present in unstable plaques (32). Its higher expression in plaques of patients with SDB may indicate that plaques of patients with SDB are more angiogenic and possibly prone to instability.

Furthermore, it is worthy of noting that dividing patients according to symptomatic if they had symptoms related to the presence of carotid plaque or asymptomatic if they did not have related symptoms (33), regardless of SDB, revealed that most of the markers investigated did not differ significantly between the groups. However, two inflammatory markers were nearly significant; neutrophil elastase—possibly indicating the presence of mature neutrophils, and lower expression of CD36+ macrophages. Therefore, we subdivided the patients into four sub-groups: non-SDB/asymptomatic; non-SDB/symptomatic; SDB/asymptomatic; and SDB/symptomatic (Table 5; Figure 1). Symptomatic patients with co-existent SDB had the highest levels of 3-NT and intracellular lipids, and the lowest SMC-actin compared with all other groups. Although the sample size is small in each sub-group, the major contribution of SDB is evident, but the contribution of being symptomatic cannot be ruled out.

Our study has several limitations that should be taken into consideration. First, the study population was relatively small. This may explain the fact that some of the between-group differences did not reach statistical significance. However, it should also be acknowledged that these experimental procedures for identifying such markers on a large scale are very tedious and time-consuming, not to mention the budget. Second, we used the Watch-PAT ambulatory device to stratify patients into SDB and non-SDB groups, rather than polysomnography. Although extensively validated against polysomnography, using the Watch-PAT might introduce some inaccuracies in identifying cases of mild SDB. However, in view of the importance of IH in inducing oxidative stress, we used ODI to classify patients into SDB and non-SDB. This measure was used in previous studies for similar classification (44). Third, 40% of the patients had COPD. This could mask the effects of the IH in patients with SDB, rendering them to a mixed intermittent and sustained hypoxia. Yet, since both groups had the same proportion of COPD while only the SDB group was associated with IH, the contribution of SDB appears substantial. Additionally, the association between carotid plaques and COPD was previously documented. Bukliosaka-Ilievska et al. (30) reported that 65% of patients with COPD had carotid plaques, and in a meta-analysis conducted by Ambrosino et al., COPD was significantly associated with subclinical atherosclerosis, particularly with a significantly thicker carotid IMT. Moreover, the prevalence of plaques was two times as high as compared with non-COPD controls, making COPD by itself a risk factor for the development of carotid plaques (31), likely due to the sustained hypoxia they experience. Collectively, making COPD a potential risk factor for carotid plaque development.

### Mechanisms Associated With Carotid Plaque Formation and Vulnerability in SDB

The risk factors promoting carotid artery disease are those identified for other types of heart disease, such as age, smoking, hypertension, hypercholesterolemia, obesity, sedentary lifestyle, family history of atherosclerosis, coronary artery disease, and most likely COPD. The formation, development, vulnerability, and rupture of plaques is a complex and multifactorial process involving many atherosclerotic pathways. Atherosclerosis is self-perpetuating due to positive feedback between plaque growth and hypoxic stress (45). It involves fundamental mechanisms, such as oxidative stress and inflammation and, thus, can promote angiogenesis *via* signaling pathways, such as hypoxia inducible factor-1 (HIF-1) and its downstream genes, such as vascular endothelial growth factor (VEGF) (46). In addition to the hypoxic stress characterizing the growing plaques, the carotid plaques of patients with SDB are exposed to an additional hypoxic burden through the IH and, thus, exacerbating various inflammatory and oxidative stress pathways (2).

## Conclusion

Plaques of patients with SDB were shown to express several markers associated with increased plaque vulnerability and rupture. The levels of 3-NT, the nitro-oxidative stress marker, and the levels of intracellular lipids were higher. Conversely, SMCs, which are considered to have a beneficial role in atherosclerotic plaque stabilization, by producing collagen and thereby contributing to the thickening of the fibrous cap, were decreased. Similarly, the increased CD31expression observed in SDB representing endothelial cell proliferation may potentially indicate that neovasculogenesis which is prevalent in unstable plaques, was also higher in SDB. Collectively, these markers indicate that carotid plaques of patients with SDB are at a higher risk of being atherogenic and vulnerable as compared with those of patients with non-SDB. Moreover, a selected group of carotid-associated symptomatic patients with co-existent SDB might be at the highest risk for plaque instability and rupture among all patients. It is, thus, conceivable that patients with SDB could be at a higher risk for stroke or worse prognosis as compared with non-SDB. On the other hand, the mortality data on SDB with stroke are scarce (33), it might be considered that in some instances, similarly to the heart, some of the patients with SDB might be protected from a more fatal brain injury due to ischemic preconditioning and development of collateral arteries, as previously shown in patients with OSA with coronary occlusion (47, 48).

## Data Availability Statement

The raw data supporting the conclusions of this article will be made available by the authors, without undue reservation.

## Ethics Statement

The studies involving human participants were reviewed and approved by the Local Human Rights Committee of RAMBAM (RMB-0175-14) Medical Center, according to the declaration of Helsinki. The patients/participants provided their written informed consent to participate in this study.

## Author Contributions

LL and AH participated in the conceptual framework of the project. AH recruited the patients and performed an endarterectomy. ES-O performed endarterectomy and organized patients' data. LL analyzed and interpreted the data, drafted, and edited the manuscript. All authors approved the final version of the manuscript.

## Funding

This study was supported by the Guzik Foundation USA.

## Conflict of Interest

The authors declare that the research was conducted in the absence of any commercial or financial relationships that could be construed as a potential conflict of interest.

## Publisher's Note

All claims expressed in this article are solely those of the authors and do not necessarily represent those of their affiliated organizations, or those of the publisher, the editors and the reviewers. Any product that may be evaluated in this article, or claim that may be made by its manufacturer, is not guaranteed or endorsed by the publisher.

## Abbreviations

SDB: sleep disordered breathing
OSA: obstructive sleep apnea
IH: intermittent hypoxia
3-NT: 3-nitrotyrosine
SMCs: smooth muscle cells
SMC-actin: smooth muscle cell-actin
IMT: intima media thickness
FMD: flow mediated dilation
CVD: cardiovascular disease
CVA: cerebrovascular accident
CD: cluster of differentiation
COPD: chronic obstructive pulmonary disease
FMD: flow mediated dilation
MI: myocardial infarction
ODI: oxygen desaturation index
AHI: apnea-hypopnea index
RDI: respiratory disturbance index
SaO_2_: oxygen saturation
Mean Oxy.: mean oxygen
Min. Oxy.: minimal oxygen
CPAP: continuous positive air pressure
HIF: hypoxia inducible factor
VEGF: vascular endothelial growth factor.
